# How dolphins see the world: A comparison with chimpanzees and humans

**DOI:** 10.1038/srep03717

**Published:** 2014-01-16

**Authors:** Masaki Tomonaga, Yuka Uwano, Toyoshi Saito

**Affiliations:** 1 Primate Research Institute, Kyoto University, Kanrin, Inuyama, Aichi 484-8506, JAPAN; 2 Port of Nagoya Public Aquarium, Minato, Nagoya, Aichi 455-0033, JAPAN

## Abstract

Bottlenose dolphins use auditory (or echoic) information to recognise their environments, and many studies have described their echolocation perception abilities. However, relatively few systematic studies have examined their visual perception. We tested dolphins on a visual-matching task using two-dimensional geometric forms including various features. Based on error patterns, we used multidimensional scaling to analyse perceptual similarities among stimuli. In addition to dolphins, we conducted comparable tests with terrestrial species: chimpanzees were tested on a computer-controlled matching task and humans were tested on a rating task. The overall perceptual similarities among stimuli in dolphins were similar to those in the two species of primates. These results clearly indicate that the visual world is perceived similarly by the three species of mammals, even though each has adapted to a different environment and has differing degrees of dependence on vision.

Because dolphins have adapted to an underwater environment, they have developed a perceptual system that differs considerably from that of terrestrial mammals such as primates. One strikingly different aspect of the perceptual system of dolphins is echolocation^1^,^2^,^3^. They can recognise shapes, materials, and the texture of objects using this form of biological sonar. Many echolocation studies on cetaceans have been conducted both in the laboratory and in the wild^4^,^5^,^6^,^7^,^8^,^9^. A few studies have investigated dolphins' ability to use cross-modal integration through vision–echolocation matching^4^,^5^,^6^,^7^. In these studies, dolphins were very accurate in matching three-dimensional complex objects using information gathered via echolocation. On the other hand, these results indirectly suggest that dolphins may also visually discriminate complex objects. Dolphins (e.g., bottlenose dolphins) have poorer in-air and underwater visual acuity (12.6 min of visual angle from a distance of 2.5 m) than that of primates^10^. Nevertheless, they still visually recognise and discriminate human gestural signs^11^,^12^,^13^, mirror images of themselves^14^,^15^, numbers of objects^16^, three-dimensional objects^4^,^17^, and two-dimensional forms^17^,^18^. Moreover, researchers have used visual stimuli to study the basic features of the vision and various cognitive abilities of dolphins^11^,^12^,^13^,^14^,^15^,^16^,^17^,^18^. However, only a few systematic studies on visual perception in dolphins have been conducted^6^, and the visual stimuli used in the previous experiments were chosen not on the basis of detailed knowledge of dolphins' perception of visual shapes but, seemingly, on the basis of extrapolation from human (or primate) visual perception. For example, some stimuli used for vision–echolocation matching had complicated three-dimensional structures^4^,^5^,^6^,^7^. While these structures may offer “rich” cues for discrimination, and thereby facilitate cross-modal matching performance, these rich cues also make it difficult for us to identify the types of visual features utilised by dolphins for such visual (or cross-modal) discrimination. We do not fully know how dolphins perceive the visual world or whether their perception differs from that of terrestrial mammals such as primates.

In the present study, we addressed this question by testing the visual form perception of dolphins using simpler geometric forms, composed of basic features. Although three-dimensionally complex stimuli might be more naturalistic, thus ecologically valid, we used these simpler patterns to compare basic properties of the visual perception of dolphins with those of other species more systematically. Bottlenose dolphins living in the Port of Nagoya Public Aquarium have been trained on face-to-face matching tasks (Fig. 1a) using various kinds of three-dimensional junk objects, such as PET bottles and flying discs. In this study, we introduced nine, novel two-dimensional forms to this matching task. The forms each varied with respect to several features (e.g. closure, curvature, incorporation of vertical, horizontal, diagonal lines, etc.; Fig. 2). By combining these stimuli, we prepared 36 pairs for discrimination and repeatedly presented these pairs to three dolphins. Based on the error patterns for each pair, we created a confusion matrix to analyse perceptual similarity among these stimuli using multidimensional scaling. For purposes of comparison, we conducted a computer-controlled matching task with chimpanzees using the same shape stimuli (Fig. 1b). Furthermore, 20 human observers also participated in rating experiments using visual analogue scaling (VAS).

## Results

The mean accuracy rate of the three dolphins was good (84.0% ± 6.1 (*SD*)) during data-collection sessions (Fig. 3a). To evaluate consistency among the dolphins, we calculated the intraclass correlation coefficient (ICC), and the value was significantly above 0, which is considered “substantial” (ICC(2,3) = 0.634, *P* = 2.86 × 10^−4^). All dolphins exhibited similar patterns of perceptual confusion. Using these accuracy data, we conducted individual differences scaling (INDSCAL), a multidimensional scaling analysis in which each dolphin's data were weighted. The resulting two-dimensional spatial configuration of perceptual similarity is shown in Figure 4a. Multidimensional scaling analyses can visualise perceptual similarities in a two-dimensional space. As shown in Figure 4a, the dolphins perceived shapes with similar features in the same way. For instance, they grouped a circle, a D-shape, and a rectangle, along with a U and an H, as similar.

Seven chimpanzees also participated in a computer-controlled matching task. All of the chimpanzees had previous experience with these kinds of computer-controlled discrimination tasks^19^,^20^. On average, they performed a two-choice matching task very well (Fig. 3b, 92.0% ± 4.1 (*SD*)). The ICC was also significantly higher than zero (ICC(2,7) = 0.845, *P* < 0.0001), indicating very high consistency among individuals. Figure 4b shows a two-dimensional configuration of the INDSCAL results. Stimuli with similar shape features (e.g., a circle, a D-shape, and a rectangle; a U-shape, an X-shape, and an H-shape) were also grouped by chimpanzees, but a major difference from the dolphins was how they perceived the X shape. It was perceived to be similar to closed shapes with diagonal lines by both species, but it was clustered with open-ended forms (U and H) only by chimpanzees. These results were similar to previous findings^21^,^22^.

Twenty human volunteers also participated in a rating experiment in which they used visual analog scaling (VAS) to rate the similarity between two forms^22^. These ratings were used as indices of dissimilarity (Fig. 3c). The ICC was also very high (ICC (2,20) = 0.906, nearly perfect, significantly above zero, *P* < 0.0001). A two-dimensional plot of perceptual similarity based on VAS results is presented in Figure 4c. Overall, the results appeared similar to those of dolphins and chimpanzees: Closed shapes, such as a circle and a D-shape, as well as shapes consisting of only vertical and horizontal lines (U, H, and a rectangle) were visually classified as similar. Additionally, as before, several differences between the classifications made by humans and those made by the other two species were observed (e.g., triangle and double-concave).

To examine similarities and differences among species, we conducted further analyses. First, we calculated the consistency of the performance differences among species using the ICC. This value, based on averaged data from each species, was 0.885, which was significantly higher than 0 (*P* < 0.0001). These data indicate that the three species exhibited comparable patterns of perceptual similarities for two-dimensional forms. Second, although we found overall similarity among species, detailed inspections led to identification of species differences. These differences may be due to differences in the weight given to the features of each form. As shown in Figure 2, each form had multiple features. In this study, we selected seven features (vertical/horizontal line, curvature, diagonal line, closure, open end, right angle, and acute angle) and calculated the mean dissimilarity among the forms sharing the same features for each species. As Figure 5 shows, we observed no significant differences among the species for four of the seven features. On the other hand, humans perceived shapes featuring a curve as more similar than did dolphins, chimpanzees perceived open-ended shapes as more similar than did the other two species, and humans did not strongly rely on acute angles for perceptual categorisations as did dolphins and chimpanzees. These results were obtained using a two-way, repeated-measures, analysis of variance (ANOVA) for species × features interactions (*F*(12, 162) = 7.607, *P* < 0.0001).

## Discussion

The main purpose of these experiments was to clarify how bottlenose dolphins visually perceive two-dimensional forms and to compare dolphins with two species of primates, chimpanzees and humans, with respect to such perceptions. Whereas a number of previous studies have been conducted on visual perception and visual–echoic cross-modal perception in dolphins^4^,^5^,^6^,^7^,^16^,^17^,^18^, many used three-dimensional objects chosen without any systematic criteria (with a few exceptions^6^) or simply used a minimal number of two-dimensional forms. Therefore, the present study is the first to investigate visual form perception in bottlenose dolphins through a systematic manipulation of the stimuli.

To our surprise, the visual perception of bottlenose dolphins is very similar to that of primates, as evidenced by our data from two visually acute terrestrial mammalian species^21^,^22^,^23^,^24^. All three species perceived shapes sharing the same features as similar, although the weight given to each feature in determining perceptual similarity differed slightly among species. For example, dolphins and chimpanzees relied on an acute-angle feature (e.g., third quadrant of Fig. 4a and left half of Fig. 4b). Open-ended features strongly affected the chimpanzees' perceptual judgments (e.g., bottom half of Fig. 4b), whereas humans used curvature for perceptual classifications (e.g., right half of Fig. 4c). The reasons for these differences in visual perception remain unclear at this point. One strong possibility relates simply to species differences in visual form perception. Another possibility is the introduction of procedural differences. Indeed, the three species performed tasks that differed in terms of task requirements (matching vs. rating) and stimulus size. In previous studies, the patterns underlying the perceptual similarities among geometric forms identified by humans differed in matching and rating tasks^22^, although the responses to these tasks were significantly positively correlated. The current results might have been particularly affected by stimulus size as it is well known that attention to global versus local features is dependent on stimulus size. Among humans and chimpanzees, attention to global features increases as the size of the stimulus decreases^25^,^26^,^27^. In the present study, humans were shown rather small shapes to rate perceptual similarity; these shapes were 30–40% smaller than the stimuli presented to dolphins and chimpanzees (as measured by visual angles). This size reduction might have led humans to rely more on a global feature such as closure. Although the physical size of the stimuli used for dolphins and chimpanzees differed, the sizes by visual angle were comparable (8–10 degrees). Previous studies on visual form perception in chimpanzees found very similar patterns of results using stimulus sets that were similar or smaller in size: a circle, a D-shape, and a rectangle; an H and an X; and shapes with acute angles (alphabet letters M, W, N, and A) were perceived as similar^22^,^23^. Thus, the similarity in visual form perception between chimpanzees and dolphins found in the present study may remain robust despite differences in the sizes of stimuli.

In the present experiment, we used “two-dimensional” stimuli for dolphins. However, these stimuli were not actually two-dimensional. Instead, they were made of PVC tubing (see the Methods section) and thus offered some depth information. Although the depth dimension presented no cues for purposes of discrimination, it is possible that the dolphins utilised the depth cues provided by stereopsis and motion parallax. Indeed, three-dimensional information helps humans match novel objects^28^. The effect of three-dimensionality on the visual form perception of dolphins should be examined further by future research.

Overall, dolphins perceive the visual world in a fashion that is similar to the way in which primates view the visual world. Avian species, such as pigeons, also share the basic features of their perception of visual forms with primates^24^,^29^,^30^, even though there are substantial differences between humans and pigeons in the global–local processing of visual stimuli^31^,^32^. Blough tested pigeons on letter discrimination and found an overall similarity with human data^24^, reporting a positive correlation between pigeon and human data (*r* = 0.68). The present study found a positive correlation between the perceptions of dolphins and chimpanzees (*r* = 0.67, *P* = 8.81 × 10^−6^), chimpanzees and humans (*r* = 0.47, *P* = 0.008), and dolphins and humans (*r* = 0.38, *P* = 0.021, using Holm's corrections). Although our data reflected weaker correlations than did Blough's data on pigeons and humans, our results suggest that the fundamental processes underpinning visual perception are shared across these three orders (primates, cetaceans, and avians). Birds and primates live in air environments, whereas cetaceans have adapted to underwater environments. However, despite the adaptations required for such different environments, these species perceive the world in fundamentally similar ways.

On the other hand, there are still differences in how different species perceive visual environments. Dolphins have poorer visual acuity than do the other species^10^ as well as very limited colour vision^33^. Moreover, pigeons rely more on local visual features than do primates^31^,^32^. However, our results provide additional basic, but important, information for comparative dolphin cognition studies^13^,^34^,^35^,^36^,^37^. Based on an understanding of the fundamental similarities and possible differences in the visual perception of dolphins and that of other species, we can examine the cetacean mind in greater detail.

## Methods

### Dolphin experiment

#### Participants

Three adult male bottlenose dolphins (*Tursiops truncates*) (Peace, Tino, and Eagle) participated in the study (see Fig. 4a). They were all wild-born and had lived in the Port of Nagoya Public Aquarium (PNPA) in Nagoya City, Aichi, Japan, for approximately 6 years at the time of the study. Their estimated ages ranged from 9 to 12 years, and they lived as a group in a pool (elliptical shape, 16 m × 11 m and 6.5 m in depth). They usually received four 15-minute sessions of husbandry, performance, and cognitive training–including matching-to-sample tasks–per day^13^. However, they did not participate in public performances during the study period. The participants were fed approximately 9 kg of fish during the training sessions, which were conducted by several trainers. The experimental procedure for the dolphins was approved by a PNPA committee and adhered to the *Ethical Guidelines for the Conduct of Research on Animals by Zoos and Aquariums* issued by the World Association of Zoos and Aquariums (WAZA), the *Code of Ethics* issued by the Japanese Association of Zoos and Aquariums (JAZA), and the Japanese *Act on Welfare and Management of Animals*.

#### Stimuli

Stimuli used in the dolphin experiment are shown in Figure 2. Each stimulus was 20 cm × 20 cm, made of PVC (polyvinyl chloride) tubing (2.5 cm in diameter), and covered with yellow waterproof vinyl tape. When the dolphins viewed the stimuli from the water (approximately 1 m from the stimulus), they had approximately 10 degrees of visual angle. These stimuli were made of three-dimensional materials, but the third dimension was not manipulated; thus, we regarded these as two-dimensional stimuli. As shown in Figure 2, these stimuli were composed of several simple features. Based on previous studies, we chose seven features to distinguish these forms from one another. The features included vertical/horizontal lines, curvature, diagonal lines, closure, open endedness, right angles, and acute angles. There were other critical features related to form perception, but features that appeared in fewer than three stimuli were omitted from analyses (such as X or T junctions).

#### Procedure

**Figure 1:**
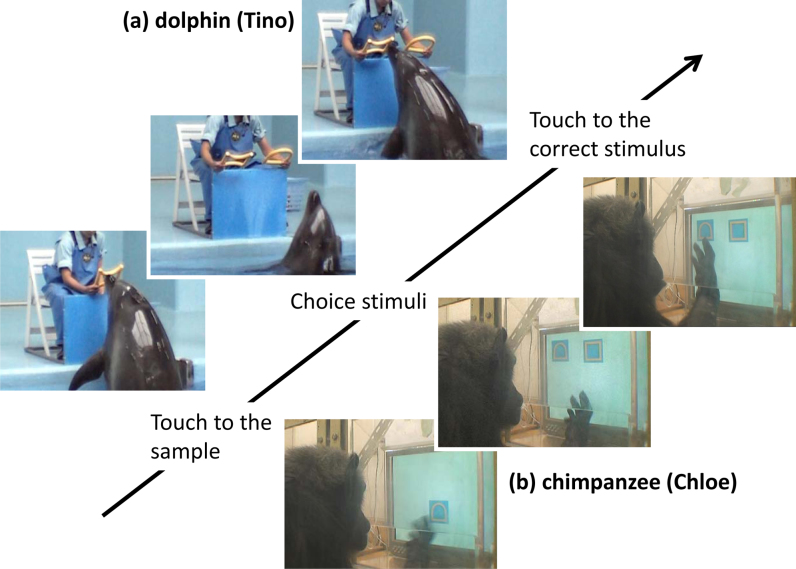
Examples of trials for dolphins and chimpanzees. (a) Tino, a bottlenose dolphin, performed face-to-face delayed matching to sample. (b) Chloe, a chimpanzee, performed computer-controlled delayed matching to sample. Note that stimulus sizes differed between species, but that visual angles were considered comparable. Photo courtesy of Masaki Tomonaga and Yuka Uwano.

**Figure 2:**
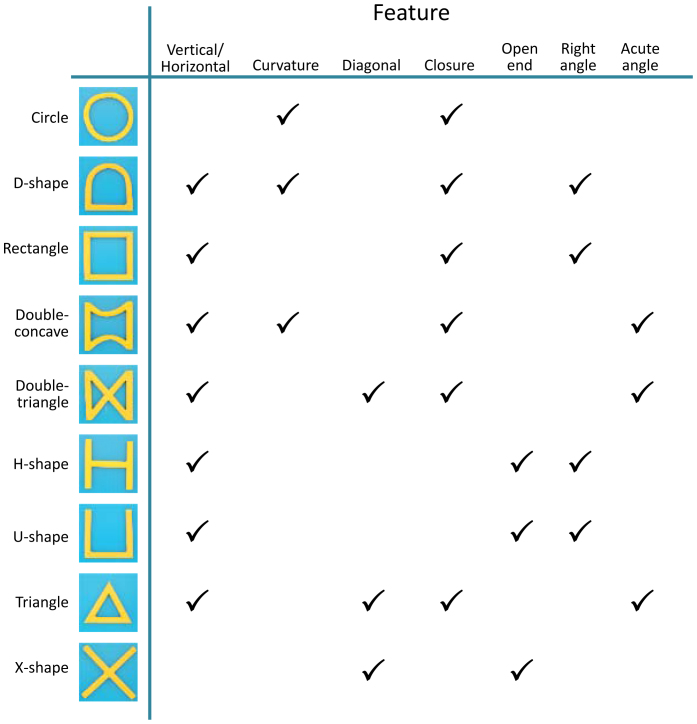
Stimuli and their related features used in the study. Right area shows the features contained within each stimulus. The study included seven distinct features.

We tested perceptual similarity using a face-to-face matching-to-sample task (Fig. 1a). Experiments were conducted in the air at poolside. Two experimenters controlled all experimental events. One of the experimenters (E1) sat behind a light blue plastic board (60 cm wide × 60 cm high). After a 10-second intertrial interval in which the dolphin remained in front of the board, E1 presented the sample stimulus by hand. The dolphin was required to touch the sample with his rostrum (“rostrum-touch”). After the rostrum-touch, E1 retracted the sample and then presented the two choice stimuli with both hands. This task is known as delayed matching. If the dolphin touched the stimulus that was identical to the sample, E1 blew a whistle, retracted both stimuli, and gave pieces of fish as a reward. If the dolphin chose the other stimulus, E1 immediately retracted the stimuli without any feedback. The other experimenter (E2) stood near the apparatus, instructed E1 about the stimulus arrangement for each trial, and recorded the dolphin's choice. Each session consisted of 10 trials, and sessions were inserted in the routine husbandry training schedules. Each dolphin participated in 1–4 sessions per day. Initially, all dolphins were trained on the matching task with three-dimensional objects, such as plastic bottles and flying discs. Using step-by-step criteria, all three dolphins engaged in this acquisition training until they showed at least 75% accuracy over 3,000 trials (3,140 trials averaged across dolphins). In this study, each dolphin showed 79% accuracy across the last 80 trials. After successfully completing acquisition training, they participated in data-collection sessions.

**Figure 3:**
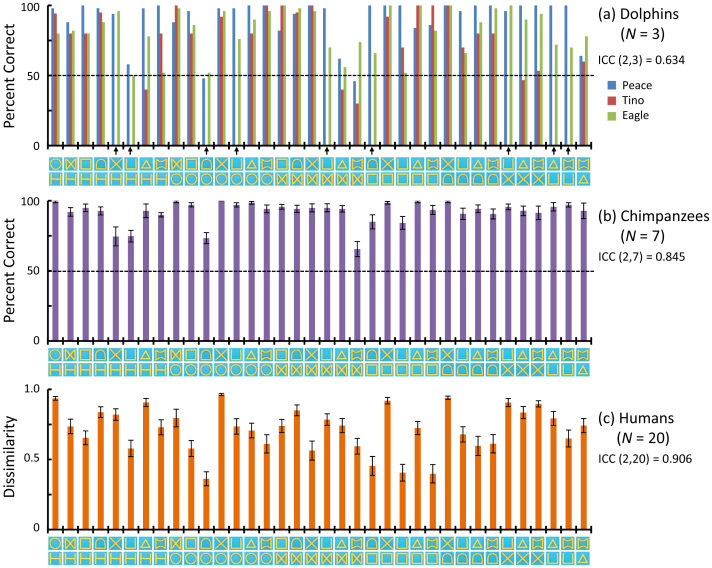
Performance on each stimuli pair across species. (a) Dolphins: Individual accuracy data are shown. Arrows on the horizontal axis indicate pairs that were not presented to Tino. (b) Chimpanzees: Average accuracy data are shown. Error bars show the standard error of the mean (SEM) across participants. (c) Humans: Average dissimilarity indices are shown. Error bars show the SEM across participants. Each graph also shows intraclass correlation coefficients (ICC). The ICC across species is 0.885.

By combining nine stimuli into pairs, we included 36 pairings in the study. However, only one pair appeared in each 10-trial session. This procedure differed from that for chimpanzees (described later) because the accuracy of the dolphins often decreased when various kinds of stimulus sets randomly appeared in one session. Dolphins Peace and Eagle received five sessions (50 trials) for each pair, receiving a total 180 sessions (1,800 trials). Due to time constraints at the Aquarium, Tino received a total of only 360 trials and was not presented with nine pairs of the 36 pairs in the study (indicated with arrows in Fig. 3a). Although Tino completed fewer trials, his performance was quite similar to that of the other two dolphins, as shown in the high intraclass correlation coefficient (ICC(2,3) = 0.634). Correlation coefficients comparing the accuracy of Tino with that of the two other dolphins were 0.40 for Tino and Peace (*P* = 0.041) and 0.52 for Tino and Eagle (*P* = 0.015), which is comparable to that for Peace and Eagle (*r* = 0.41, *P* = 0.028, using Holm's corrections). Note that response-time data, although recorded during the study, were not used for data analyses because of considerable variance.

### Chimpanzee experiment

#### Participants

**Figure 4:**
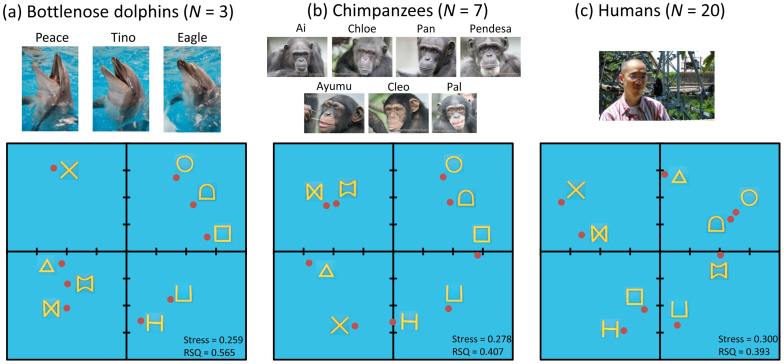
Two-dimensional solutions of multidimensional scaling analyses. Each axis ranged from −2.0 to 2.0. Each graph also showed the stress value and RSQ for evaluation of goodness of fit. (a) Dolphins, (b) Chimpanzees, and (c) Humans. Photo courtesy of Masaki Tomonaga and Yuka Uwano.

**Figure 5:**
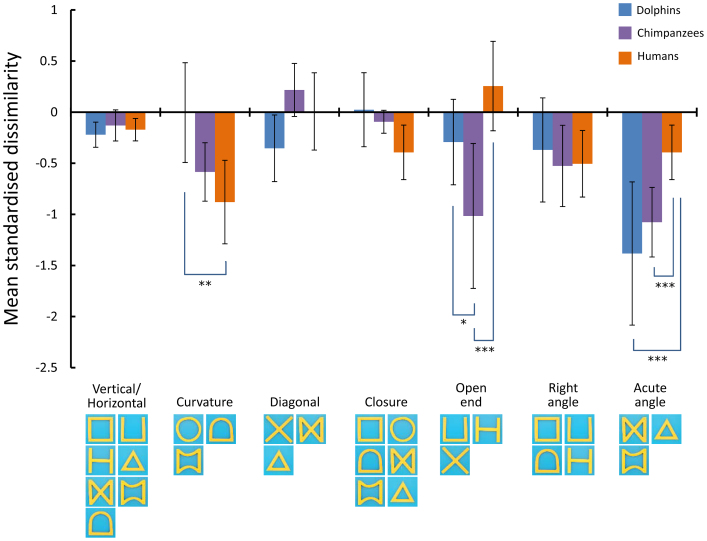
Relative contribution of each feature to perceptual similarities. Vertical axis shows the mean standardized dissimilarity (see details in the Methods section). A smaller value indicates that forms sharing those features are perceptually more similar to one another. *: *P* < 0.05, **: *P* < 0.01, ***: *P* < 0.001, multiple comparisons after repeated-measures ANOVA.

Seven chimpanzees (*Pan troglodytes*) (Ai, 33-year-old female; Ayumu, 10-year-old male; Chloe, 29-year-old female; Cleo, 10-year-old female; Pan, 26-year-old female; Pal, 10-year-old female; and Pendesa, 33-year-old female) participated in the study (see Fig. 4b). All participants lived in a social group of 14 individuals in an indoor and environmentally enriched outdoor compound (770 m^2^) at the Primate Research Institute, Kyoto University (KUPRI), Japan^38^. They were not food-deprived and were fed fruits, vegetables, and primate chow three times each day during the study. They had previously engaged in various kinds of computer-controlled perceptual and cognitive tasks, including those involving matching to sample^19^,^21^,^22^,^39^,^40^,^41^,^42^,^43^,^44^. As a result, all chimpanzee participants were already familiar with generalised identity-matching tasks at the beginning of the present experiments; thus, we did not need to conduct any acquisition training for this group. The mean accuracy of all participants was 87.3% ± 6.6 (*SD*) in the first session. The care and use of the chimpanzees adhered to the 3rd edition of the *Guide for the Care and Use of Laboratory Primates* issued by KUPRI in 2010, which is compatible with the guidelines issued by the National Institute of Health in the United States of America. The research design was approved by the Animal Welfare and Animal Care Committee of KUPRI and by the Animal Research Committee of Kyoto University. All procedures adhered to the Japanese *Act on Welfare and Management of Animals*.

#### Apparatus and stimuli

Experimental sessions were conducted in a booth (1.8 × 2.15 × 1.75 m) in the experimental room adjacent to the chimpanzee facility. Each chimpanzee came to the booth via an overhead walkway connecting the facility and the booth. A 17-inch LCD monitor (1280 × 1024 pixels, pixel size: 0.264 mm × 0.264 mm) with a touch panel was installed on the wall of the booth (see Fig. 1b). Viewing distance was approximately 40 cm. The food reward was delivered via a universal feeder. All equipment and experimental events were controlled by computer. Stimuli used for the chimpanzees were colour photographs of the stimuli used for the dolphins. Each stimulus was 210 pixels × 210 pixels (55 mm × 55 mm, approximately 8 deg of visual angle) and was presented in yellow against a light blue background (see Fig. 1b).

#### Procedure

We used a delayed matching task with the chimpanzee participants (Fig. 1b). Each trial began with the presentation of a blue square (26 mm × 26 mm) at the bottom centre of the monitor. When the chimpanzee touched this square twice, a sample stimulus appeared at one of six (three columns × two rows) predetermined locations. When the chimpanzee touched the sample, it immediately disappeared, followed by the presentation of the two choice stimuli. The configuration of the stimuli was randomly changed from trial to trial. If the chimpanzee touched the choice stimulus identical to the sample, all stimuli disappeared, followed by the sound of a chime and the presentation of a food reward (a small piece of apple or raisin). If the chimpanzee touched the other stimulus, a buzzer sound was presented as error feedback. Following an error trial, a modified version of a correction procedure was used in which only the correct choice stimulus was presented (for correction only; not used in data analyses). This procedure was used to prevent inappropriate runs of error trials. The intertrial interval was 2 seconds. In contrast to the sessions with dolphins, sessions with the chimpanzees consisted of 36 trials, during which all 36 pairs appeared once. Each chimpanzee received 20 sessions; therefore, we collected data from 20 trials for each pair. As in the dolphin trials, response-time data were not used for analyses although they were recorded at the time.

### Human experiments

#### Participants

Twenty PNPA volunteers participated as a group in the rating experiment. All participants had normal or corrected-to-normal vision. The purpose of and procedure for rating were explained orally by the experimenter, and informed consent was obtained from all participants. All protocols were approved by the Human Research Ethics Committee of KUPRI.

#### Procedure

We used visual analog scaling (VAS) in the experiment with humans^22^. Participants sat in a visitor room and received a sheet on which pictures of the 36 stimuli pairs were printed. Each stimulus was 7 mm × 7 mm (approximately 3 deg of visual angle). Participants were instructed to judge the similarity of the stimulus pair and make a mark on an adjacent horizontal line. The rating for “most dissimilar” was placed on the left end of the line, and the mark for “most similar” was placed on the right end of the line. The sheets were scanned and converted to digital image files. Using customised application software, dissimilarity was calculated based on the spatial location of the check mark.

### Data analysis

#### Dissimilarity index

For dolphins and chimpanzees, the dissimilarity index for each pair was defined as the absolute value of the percent of correct choices subtracted by 50% (chance level). This index ranged from 0 (most similar) to 50 (most dissimilar). For humans, the relative distance of the mark from the right was used as the dissimilarity index, which also ranged from 0 to 1.

#### Inter-observer variability

To evaluate similarities in the performances of participants, we calculated the ICC using random-effects models (i.e., ICC(2, *n*); *n* designates the number of participants). These values were calculated using SPSS 19.0 J and statistically tested with a null hypothesis of ICC = 0. Furthermore, using dissimilarity data averaged across participants, we also calculated the ICC (using a mixed effects model) for evaluating inter-species variability.

#### Multidimensional scaling analysis

To understand the spatial configuration of perceptual similarities among stimuli, we conducted multidimensional scaling analyses for each species using INDSCAL. This method yielded spatial representations for the stimuli as well as weights for each dimension of this representation for each observer. We adopted a two-dimensional solution for the present analyses using SPSS 19.0J. Obtained representations ranged from −2.0 to 2.0 for each dimension. To evaluate goodness of fit, we presented stress values and coefficients of determination (RSQ).

#### Relative contribution of features

To evaluate the relative contribution of each individual feature to the perceptual grouping, we calculated the mean percent of correct choices for pairs in which both stimuli had the same individual features. These values were standardised using means and standard deviations (referred to as standardised dissimilarity); they were then analysed with a two-way (species × features) repeated-measures ANOVA.

## Author Contributions

M.T., Y.U. and T.S. conceived the experiments; Y.U. and T.S. performed the experiments; and M.T., Y.U. and T.S. analysed the data. All authors discussed the results. M.T. wrote the paper, and all other authors commented on the manuscript.
